# Supplementary material for: Coronavirus membrane protein with a fluorescent protein tag enables tracking of virus particles in live cells
Source: bioRxiv. 2025 Dec 30:2025.12.29.696688. Preprint. [Version 1] doi: 10.64898/2025.12.29.696688 (PMC12776301; doi:10.64898/2025.12.29.696688)
Supplement: 1 [file NIHPP2025.12.29.696688v1-supplement-1.pdf]

bioRxiv preprint doi: https://doi.org/10.64898/2025.12.29.696688; this version posted December 30, 2025. The copyright holder has placed this preprint (which was not certified by peer review) in the Public Domain. It is no longer restricted by copyright. Anyone can legally share, reuse, remix, or adapt this material for any purpose without crediting the original authors.

FIGURE LEGENDS SUPPLEMENT

Fig. S1. M-GFP used to track the progression of MHV infection in live cells. L2 cells were infected with M-GFP MHV. Nuclei were labeled with Hoechst. Cells were imaged by confocal. Z-stacks were collected every 2 minutes from 5-10 hpi. Movie is a 3D rendered view from the perspective of looking down on the cells from above. Green=GFP; blue=nuclei (Hoechst).

Fig. S2. M-GFP protein remains intact when expressed. (A) L2 cells were transfected with untagged WT M or GFP; M-GFP with a V5 tag at the amino end; GFP or M-GFP with a V5 tag at the carboxy end. At 24 h after transfection cell lysates were immunoblotted and probed with antibodies against GFP or V5. When probed with anti-GFP and anti-V5, the 5’ V5 M-GFP and the 3’ V5 M-GFP show bands 37kDa and 50 kDa (lanes 5 and 7). (B) L2 cells were transfected with M-GFP with the amino V5 tag

bioRxiv preprint doi: https://doi.org/10.64898/2025.12.29.696688; this version posted December 30, 2025. The copyright holder has placed this preprint (which was not certified by peer review) in the Public Domain. It is no longer restricted by copyright. Anyone can legally share, reuse, remix, or adapt this material for any purpose without crediting the original authors.

(top row) or amino V5 tag (bottom row) (B) At 24 hours after transfection, cells were immunolabeled for WT M and V5, imaged by confocal. Typical fields are shown selected from at least 3 fields acquired from each of 2 independent experiments. White=nuclei (DAPI); green=M-GFP; red=WT M; blue=V5. Scale bar = 10 μm.

Fig. S3. M-GFP allows imaging of assembled MHV virus trafficking at and near the plasma membrane. 17C1 cells were infected with M-GFP MHV then imaged live by TIRF microscopy at 6-7hpi. TIRF was collected in the GFP channel and overlaid on differential interference contrast, still image. Timelapse images were collected for up to 2.5 minutes per field. A typical timelapse is shown, selected from at least 5 datasets taken from each of 3 independent experiments.

Fig. S4. Tracking of virus at and near the plasma membrane in 17Cl1 infected cells. Timelapse images collected by TIRF (as described in Fig. S3) were analyzed by Nikon Elements particle tracking module. Movie shows tracks detected from 60 seconds of data collection.

SUPPLEMENTAL

MATERIALS AND METHODS

Microscopy. For live cell z-stack, time-lapse images, L2 cells were grown in an 8-well Ibidi chambered coverglass. After infection with M-GFP MHV, nuclei were labeled with Hoechst. Images were collected on a Nikon AX R confocal, using a 60x, oil immersion, 1.4 N.A. lens. The Nikon Ti-2 microscope base was equipped with a Tokai-Hit stage-top incubator which was maintained at 37°C and 5% CO_2. Cells were imaged from 5-10 hpi. Full z-stacks through the cells were collected every 2 minutes. Fixed immunofluorescent imaging, live cell TIRF, and particle tracking were done as described in main text.

bioRxiv preprint doi: https://doi.org/10.64898/2025.12.29.696688; this version posted December 30, 2025. The copyright holder has placed this preprint (which was not certified by peer review) in the Public Domain. It is no longer restricted by copyright. Anyone can legally share, reuse, remix, or adapt this material for any purpose without crediting the original authors.

Supplemental Material

S1, S3, S4 Movies
(only representative still images included for preprint)

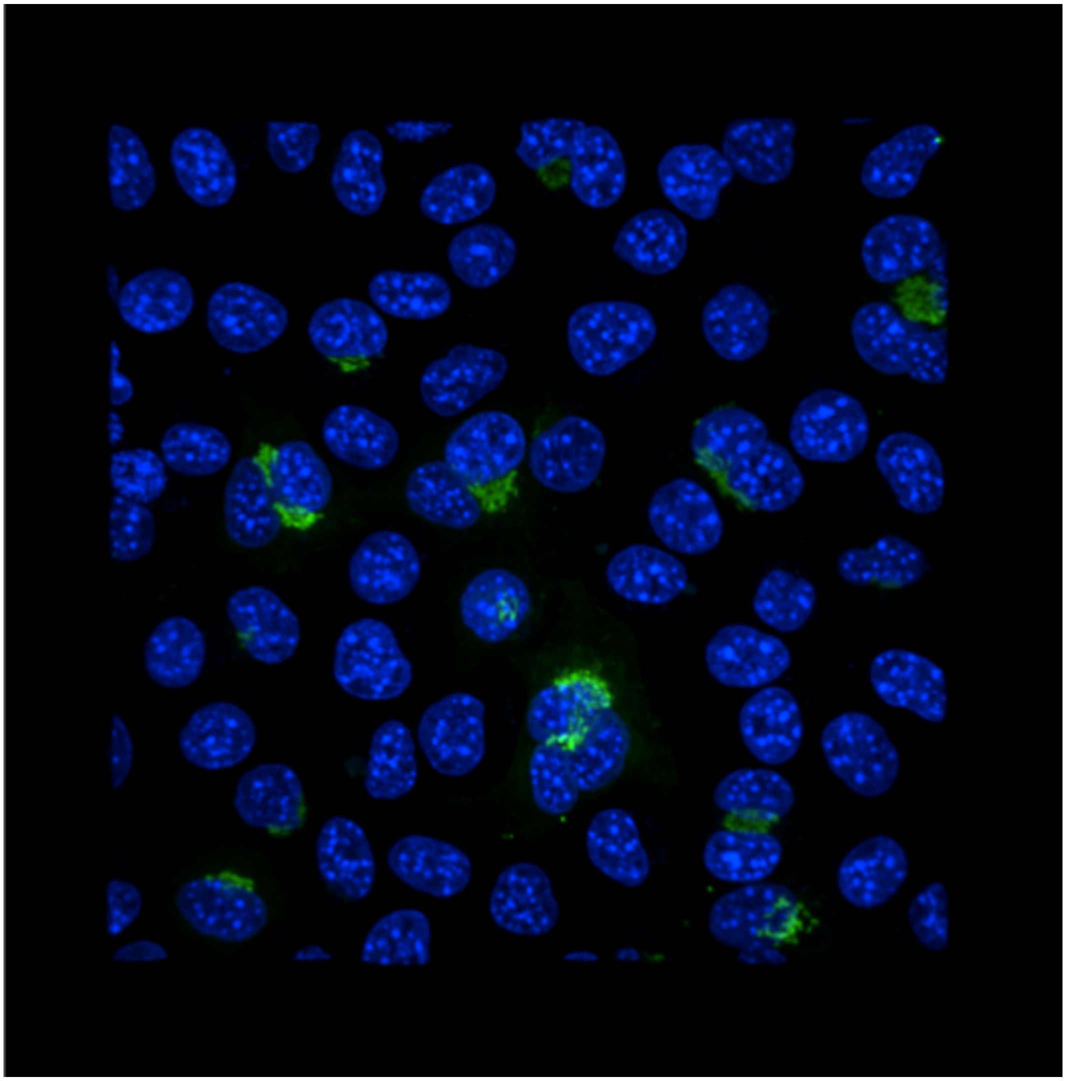

bioRxiv preprint doi: https://doi.org/10.64898/2025.12.29.696688; this version posted December 30, 2025. The copyright holder has placed this preprint (which was not certified by peer review) in the Public Domain. It is no longer restricted by copyright. Anyone can legally share, reuse, remix, or adapt this material for any purpose without crediting the original authors.

Fig. S1

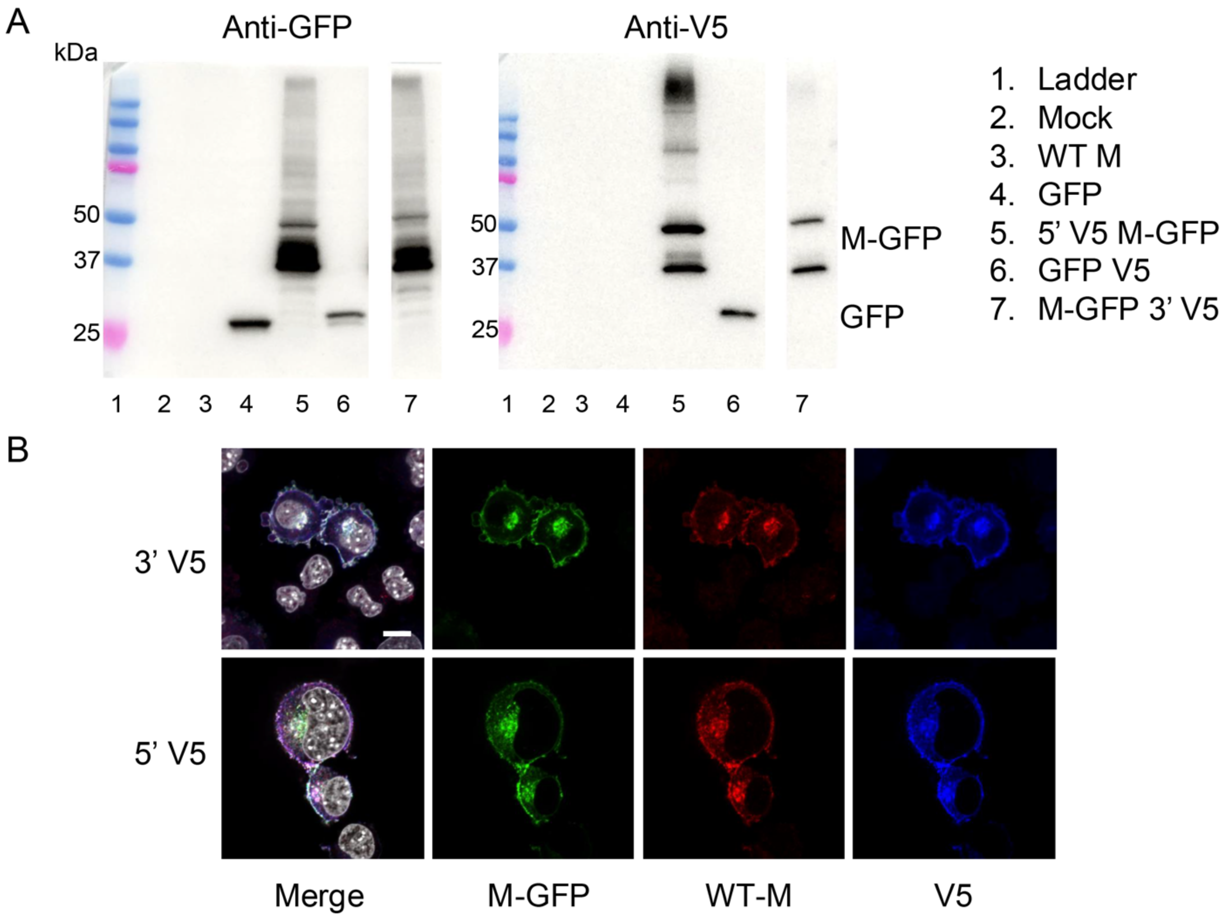

Fig. S2

bioRxiv preprint doi: https://doi.org/10.64898/2025.12.29.696688; this version posted December 30, 2025. The copyright holder has placed this preprint (which was not certified by peer review) in the Public Domain. It is no longer restricted by copyright. Anyone can legally share, reuse, remix, or adapt this material for any purpose without crediting the original authors.

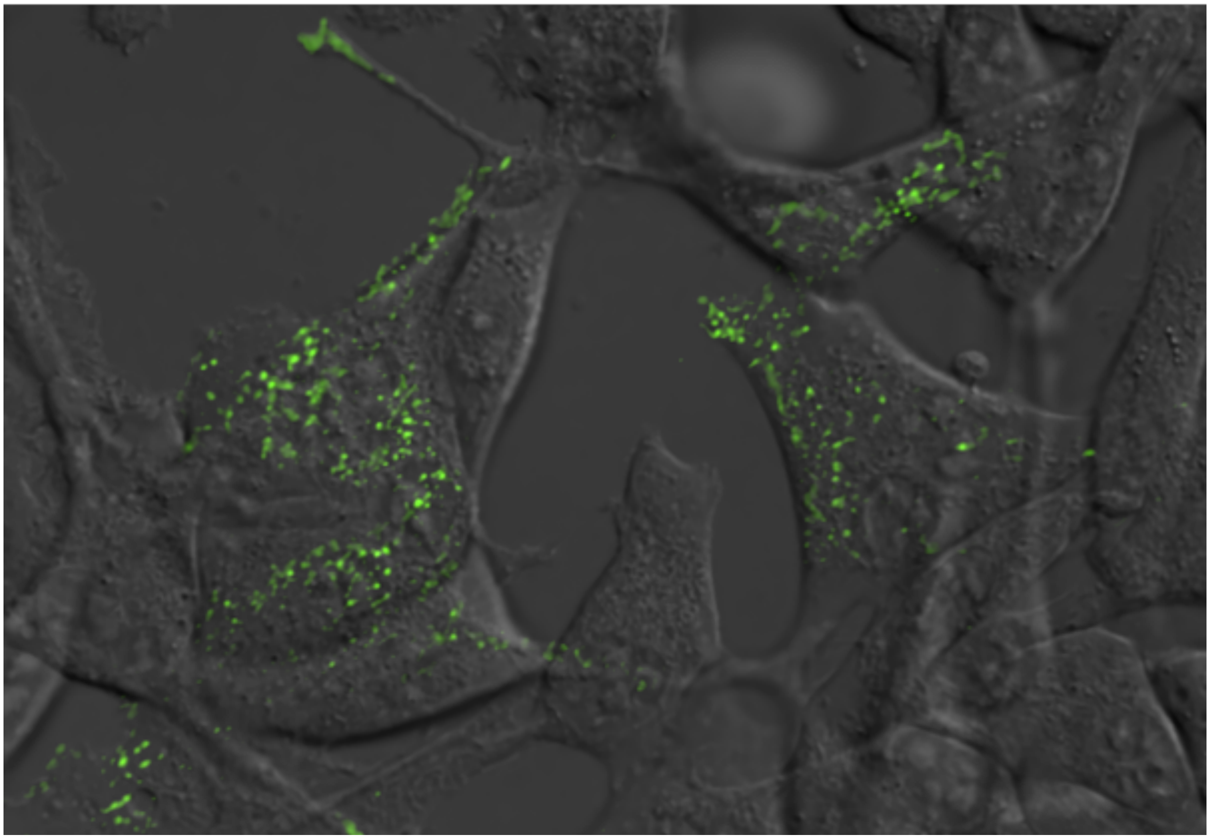

Fig. S3

bioRxiv preprint doi: https://doi.org/10.64898/2025.12.29.696688; this version posted December 30, 2025. The copyright holder has placed this preprint (which was not certified by peer review) in the Public Domain. It is no longer restricted by copyright. Anyone can legally share, reuse, remix, or adapt this material for any purpose without crediting the original authors.

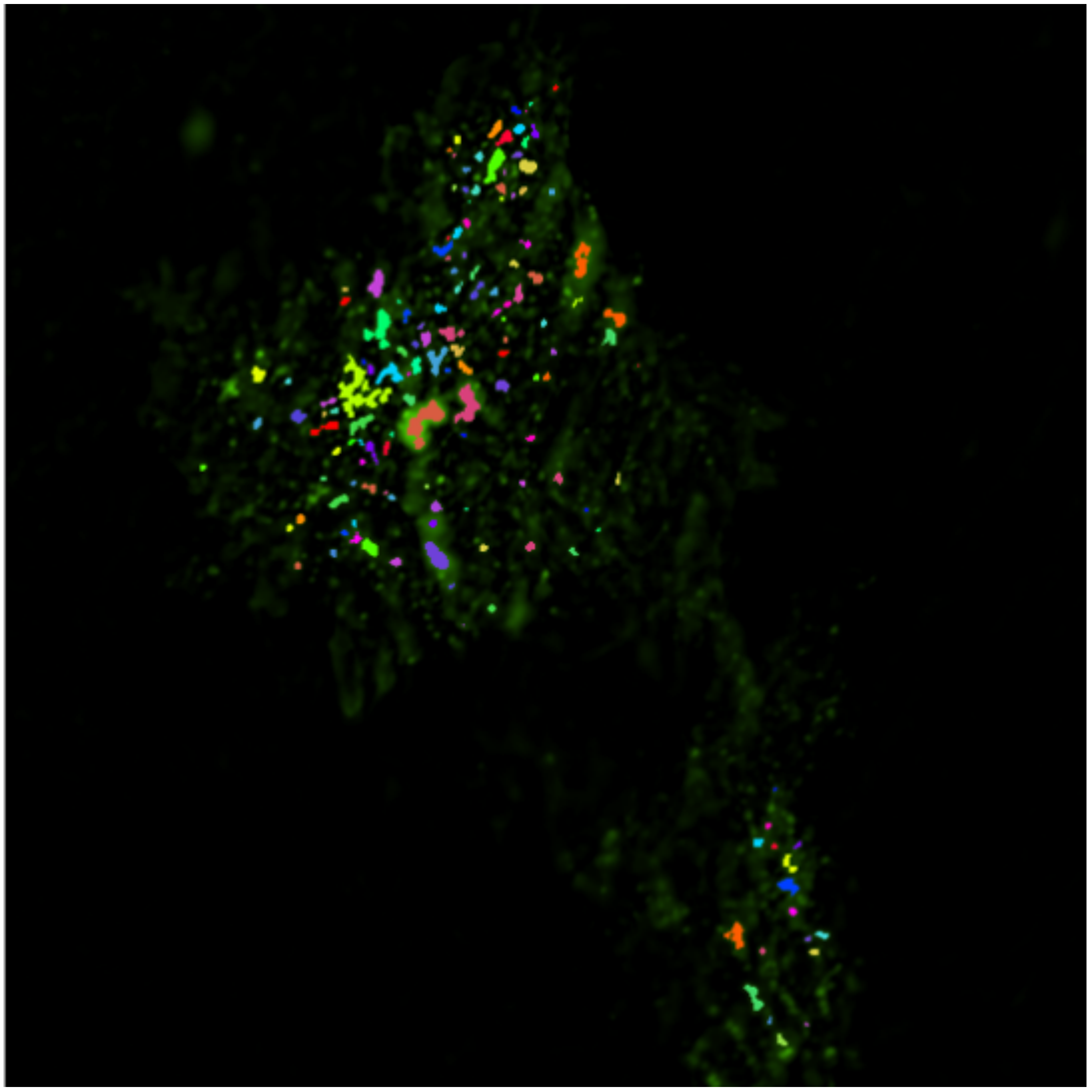

Fig. S4.
